# Comparative evaluation of screening and supplementary assays used in HCV diagnosis

**DOI:** 10.1186/1471-2334-12-S1-P65

**Published:** 2012-05-04

**Authors:** Vidya Nerurkar, Prabhupad Rath, Rashmi Khadapkar, Simi Bhatia, Bibhu Ranjan Das

**Affiliations:** 1 Super Religare Laboratories, Mumbai - 400 062, Maharashtra, India

## Background

Evaluation of serological assays for hepatitis C (HCV) antibody screening is crucial ,as supplemental testing [Nucleic Acid Testing & Recombinant Immunoblot Assay (RIBA)] to verify anti-HCV reactive results is not widely practiced, due to limited availability and high cost. The current study was undertaken from January to May 2011, to evaluate 4 serological assays for HCV diagnosis, viz-a-viz HCV RNA PCR and RIBA.

## Methods

87 patient specimens were screened for anti-HCV, using COBASe411(eCLIA, Roche, Germany), Axsym (MEIA, Abbott, Germany), HCV Qualisa (ELISA, Qualpro, India) and HCV Tridot (Rapid Immunofiltration, J. Mitra, India), and for presence of HCV RNA using COBAS TAQMAN 48 analyzer (Roche). Specimens with discrepant results were referred internationally for RIBA.

## Results

58/87 (66.7%) specimens were anti-HCV reactive. 37/58 were HCV RNA PCR positive, indicating active HCV infection. Sensitivities of COBAS, Axsym, Qualisa and Tridot were 100%, 97.3%, 72.9% and 75.7% respectively. False negative anti-HCV results, obtained by Qualisa and Tridot, were seen in 10 and 9 patients respectively and could be attributed to the synthetic peptide coating in these kits. 6 of these patients were chronic renal failure cases, on hemodialysis. All kits showed specificity of 100%. False positivity was not observed, possibly because our study group comprised of patients with suspected HCV infection.

## Conclusion

Anti-HCV assays on COBASe411 (eCLIA) and Axsym (MEIA) platforms appear reliable. The study thus highlights the importance of using recombinant antigen based tests for anti-HCV screening. A strong need to conduct larger studies for performance evaluation of anti-HCV tests in specific patient subpopulations is felt.

